# Adult *Diaphorina citri* Biocontrol Using *Hirsutella citriformis* Strains and Gum Formulations

**DOI:** 10.3390/plants12183184

**Published:** 2023-09-06

**Authors:** Servando H. Cantú-Bernal, Ricardo Gomez-Flores, Rosa A. Flores-Villarreal, Alonso A. Orozco-Flores, César I. Romo-Sáenz, Roberto Montesinos-Matías, Marco A. Mellín-Rosas, Jorge A. Sánchez-González, Orquídea Pérez-González, Patricia Tamez-Guerra

**Affiliations:** 1 Facultad de Ciencias Biológicas, Departamento de Microbiología e Inmunología, Avenida Pedro de Alba s/n, Ciudad Universitaria, Universidad Autónoma de Nuevo León, San Nicolás de los Garza 66455, Nuevo León, Mexico; servando.cantubr@uanl.edu.mx (S.H.C.-B.); ricardo.gomezfl@uanl.edu.mx (R.G.-F.); rosa.floresvll@uanl.edu.mx (R.A.F.-V.); aorozcof@uanl.edu.mx (A.A.O.-F.); cesar.romosnz@uanl.edu.mx (C.I.R.-S.); 2 Centro Nacional de Referencia de Control Biológico—CNRF, Dirección General de Sanidad Vegetal-SENASICA-SADER, Km 1.5 Carretera Tecomán—Estación FFCC, Col. Tepeyac, Tecomán 28110, Colima, Mexico; montesinosroberto1612@gmail.com (R.M.-M.); marco.mellin@senasica.gob.mx (M.A.M.-R.); antonio.sanchez@senasica.gob.mx (J.A.S.-G.)

**Keywords:** Asian citrus psyllid, improved bioinsecticide, citrus orchards

## Abstract

*Hirsutella citriformis* Speare is the only entomopathogenic fungus that has been applied to control the hemipteran *Diaphorina citri* Kuwayama. However, the use of available commercial products under field conditions is limited due to conidia’s shelf life and short environmental persistence. We have previously reported the citrus psyllid *D. citri* adults’ biocontrol potential using *H. citriformis* strains. The aim of the present study was to evaluate different formulations based on *H. citriformis* (OP-Hir-3, OP-Hir-10, and OP-Hir-12 strains) conidia and gums as additives to improve *D. citri* adults’ biocontrol, under laboratory, greenhouse, and field conditions, using *Hirsutella* gums as conidia stabilizers to improve their viability under environmental drought conditions and as insecticide. Laboratory bioassay results showed that the highest (*p* < 0.05) *D. citri* mortality was achieved using FOP-Hir-10GH (63.5%), followed by the *Hirsutella* gum control (42.2%). Under greenhouse conditions, adults’ mortality reached up to 84.6% with FOP-Hir-12 and 49.0% with *Hirsutella* gum. In addition, we applied *H. citriformis* formulations under field conditions in a commercial citrus grove located in Tecomán, Colima, México, at 21.5 °C and 73.3% relative humidity (RH) in March and 25.7 °C and 72.5% RH in October 2022 and observed 67.3% and 94.0% mortality of *D. citri* adults, respectively. *Hirsutella* gum alone showed significant insecticidal activity against *D. citri* adults. In conclusion, this study demonstrated that *Hirsutella* gum functioned as additive to *H. citriformis* conidia formulations, improving *D. citri* adults’ mortality and showing potential for this pest biocontrol in citrus orchards.

## 1. Introduction

*Diaphorina citri* Kuwayama (Hemiptera: Psyllidae), known as the Asian citrus psyllid, is the most serious threat to citrus crops worldwide. It is the vector of *Candidatus* Liberibacter spp., the causal agent of the Huanglongbing disease (HLB). *D. citri* is widely distributed in citrus growing regions, including tropical and subtropical Asia, the Middle East, Central America, South America, Mexico, and Brazil [1]. There is no cure for trees infected by this citrus-devastating disease [2,3]. Mexican citrus-producing orchards comprise 56.0% oranges (*Citrus* × *sinensis* L.) Osbeck, 33.5% lemons (*Citrus aurantiifolia* (Christm.) Swingle, and 10.5% other citrus fruits, such as grapefruit (*Citrus paradisi* Macfad) and mandarin orange (*Citrus reticulate* Blanco) [4]. HLB was detected in the Campeche and Quintana Roo Mexican states in 2002, and it has spread to all citrus-producing country areas [5]. HLB has been detected in 351 municipalities belonging to 25 Mexican citrus-producer states, where 8.3 million citrus tons are produced yearly, representing approximately 1523 million US dollars [4]. The greatest HLB damage was recorded in Colima, Nayarit, Jalisco, Michoacán, and Sinaloa commercial orchards [6]. We currently lack an effective control strategy for HLB disease, except for infection prevention methods for trees based on controlling the vector *D. citri* through chemical, biological, and cultural control, with the chemical control being the most commonly used. However, the application of excessive chemical insecticides during the last decades has led to environmental contamination, health threat, and target and non-target insects’ selective pressure and resistance development [7,8]. To prevent adverse effects from chemical insecticides, biological control using a variety of natural enemies against this psyllid has been applied [9]. 

Natural enemies reported for biological control of *D. citri* include parasitoids such as *Tamarixia radiata* Waterston (Hymenoptera: Eulophidae) and *Diaphorencytus aligarhensis* (Hymenoptera: Encyrtidae), Coleoptera, Neuroptera, and Diptera predators such as coccinellid beetles, syrphids, spiders, and lacewing larvae, as well as entomopathogenic microorganisms [9,10]. Fungi have emerged as promising entomopathogenic microorganisms, due to their potential to actively infect all *D. citri* life stages and their laboratory mass-produced feasibility. They include *Isaria* (*Cordyceps*) *fumosorosea* (Wize), *Lecanicillium lecanii* Zimm., *Beauveria* (*Cordyceps*) *bassiana* (Bals.-Criv.), *Metarhizium anisopliae*, and *Hirsutella citriformis* Speare [11,12,13]. Their main advantage is the reduction of insects’ resistance development, compared to that induced via chemical insecticides [10]. 

*H. citriformis* is the only fungus naturally found causing *D. citri* epizootics in various regions worldwide [12,14,15]. Unfortunately, conidia’s short persistence under field conditions and its shelf life compared with other entomopatogenic fungi has limited its commercial use. It has been demonstrated that entomopatogenic fungus formulations may increase active agent residual activity in the field, thus improving biocontrol of the target insect [16]. We have found that *Hirsutella* gum, in addition to protecting conidia from drought, increases *D. citri* adults’ mortality [17]. The aim of the present study was to evaluate the effectiveness of formulated conidia from three selected *H. citriformis* strains and gums against *D. citri* adults, under laboratory, greenhouse, and field conditions.

## 2. Results

### 2.1. Hirsutella citriformis Efficacy against Diaphorina citri Adults under Laboratory Conditions

**Figure 1:**
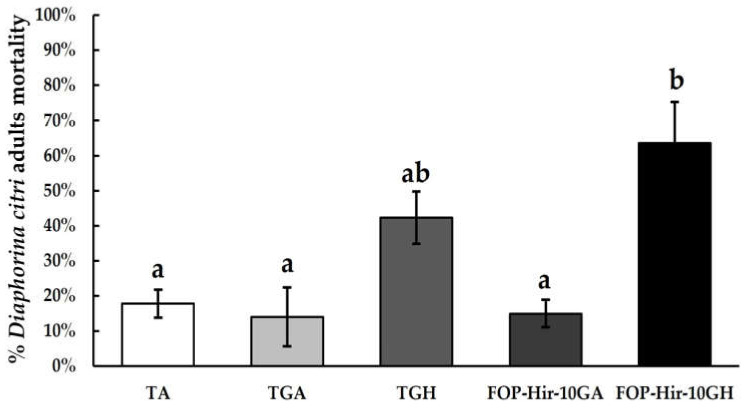
*Diaphorina citri* adults’ mortality percentages after spraying application of formulated *Hirsutella citriformis* conidia (1 × 10^7^ conidia/mL), under laboratory conditions. TA = absolute control; TGA = *Acacia* gum + vegetable oil powder control; TGH = *Hirsutella* gum + vegetable oil powder control; FOP-Hir-10GA = formulated conidia + *Acacia* gum + vegetable oil powder; and FOP-Hir-10GH = formulated conidia + *Hirsutella* gum + vegetable oil powder. The *Hirsutella* gum control (TGH) presented a mortality of 57.8%. Bars indicate the standard error (Tukey test α ˂ 0.05) and different letters indicate significant statistical differences.

The additive effect of *Acacia* and *Hirsutella* gums was included in conidia formulations. Under laboratory conditions, we showed that FOP-Hir-10GH strain-formulated conidia were the most effective at controlling *D. citri* adults after spraying application, achieving 63.5% mortality of the treated insects after 21 d application, compared with the absolute control (TA; F_4,19_ = 8.0, *p* ˂ 0.001). In addition, *H. citriformis* gum without conidia (TGH treatment) resulted in 42.2% mortality of *D. citri* adults, thus demonstrating *H. citriformis* gum’s direct toxicity to *D. citri* adults. TGA and FOP-Hir-10GA treatments did not show significant differences, compared with TA (Figure 1). FOP-Hir-10GH treatment also showed a lower lethal time (LT_50_) of 9 d (CL_95_ = 6.2–11.7), compared with that of the TGH treatment, which was 14.7 d (CL_95_ = 12.9–16.5) (Table 1).

### 2.2. Hirsutella citriformis Efficacy against Diaphorina citri Adults under Greenhouse Conditions 

**Figure 2:**
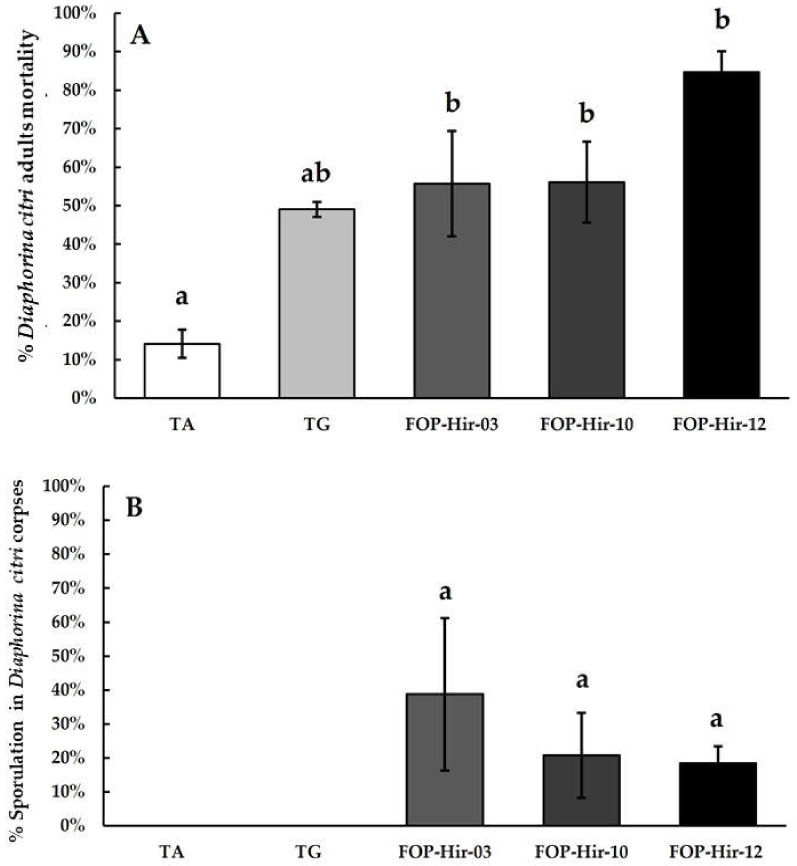
Mortality percentages and aerial mycelium development (sporulation) averages caused by formulated *Hirsutella citriformis* conidia (5 × 10^6^ conidia/mL), applied via spraying on *Diaphorina citri* adults in greenhouse bioassays. TA = absolute control; TG = control without active agent; FOP-Hir-03 = OP-Hir-3 strain formulation; FOP-Hir-10 = OP-Hir-10 strain formulation; and FOP-Hir-12 = OP-Hir-12 strain formulation. (**A**) Average mortality percentage. Bars indicate the standard error (Tukey α ˂ 0.05) and different lower-case letters indicate a significant statistical difference between treatments. (**B**) Percentage of the average development of aerial mycelium. Different letters indicate a statistically significant (Dunn’s α ˂ 0.05) difference in the percentage of sporulation of the fungus in the infected insects, using the Kruskal–Wallis test.

The efficacy evaluation of formulated *H. citriformis* conidia in greenhouse experiments revealed significant differences between the formulations and the absolute control (F_4,19_ = 8.6; *p* ≤ 0.05). The highest mortality of *D. citri* adults was observed with the OP-Hir-12 formulation, where fungi infected and killed 84.6% of treated insects. FOP-Hir-03 and FOP-Hir-10 formulations showed 55.7% and 55.7%, respectively, mortality in *D. citri* adults (Figure 2A). 

In addition, the TG treatment resulted in 49.0% mortality of *D. citri* adults, showing a similar effect than that observed in laboratory bioassays (Figure 2A). We did not find significant (*H* (4) = 8.226, *p* = 0.0836) differences in sporulation corpses among treatments. Since the data did not show normal distribution, we used the Kruskal–Wallis analysis. The largest aerial mycelia development by *D. citri* cadavers was caused by the FOP-Hir-03 treatment, where 38.7% mycosed cadavers were detected, whereas FOP-Hir-10 and FOP-Hir-12 treatments resulted in 20.8% and 18.4% mycosed cadavers, respectively (Figure 2B). 

### 2.3. Hirsutella citriformis Efficacy against Diaphorina citri Adults under Field Conditions 

The *D. citri* adults’ mortality from the application of formulated *H. citriformis* conidia on a citrus orchard was significantly higher compared to the absolute control during the March 2022 assay. FOP-Hir-12 caused the highest mortality, where fungi infected and killed 67.3% of *D. citri*-treated adults (F_4,14_ = 44.2; *p* ≤ 0.05). FOP-Hir-03 and FOP-Hir-10 treatments resulted in 57.0% and 54.2% mortality of *D. citri* adults, respectively. 

**Figure 3:**
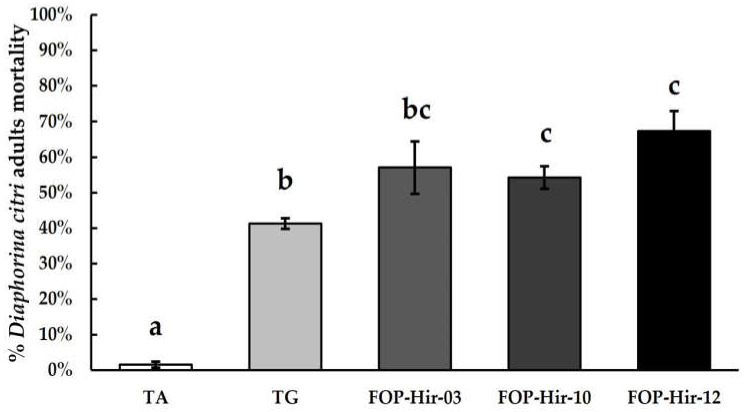
*Diaphorina citri* adults’ mortality percentages after *Hirsutella citriformis*-formulated conidia application at 5 × 10^6^ conidia/mL via spraying under field conditions. Absolute control = TA; *Acacia* gum + *Hirsutella* gum + vegetable oil powder control = TG; FOP-Hir-03 conidia + *Hirsutella* gum + vegetable oil powder = OP-Hir-03-formulated conidia; FOP-Hir-10 conidia + *Hirsutella* gum + vegetable oil powder = OP-Hir-10-formulated conidia; FOP-Hir-12 conidia + *Hirsutella* gum + vegetable oil powder = OP-Hir-12-formulated conidia. Bars indicate the standard error (Tukey α ˂ 0.05), and different lower-case letters indicate a significant statistical difference between treatments.

*Hirsutella* gum alone showed a similar effect to that observed in laboratory and greenhouse bioassays, causing 41.2% mortality among *D. citri*-treated adults (Figure 3). During the experimental period, it was not possible to detect *H. citriformis* aerial mycelia development on *D. citri* cadavers collected from treated field areas.

**Figure 4:**
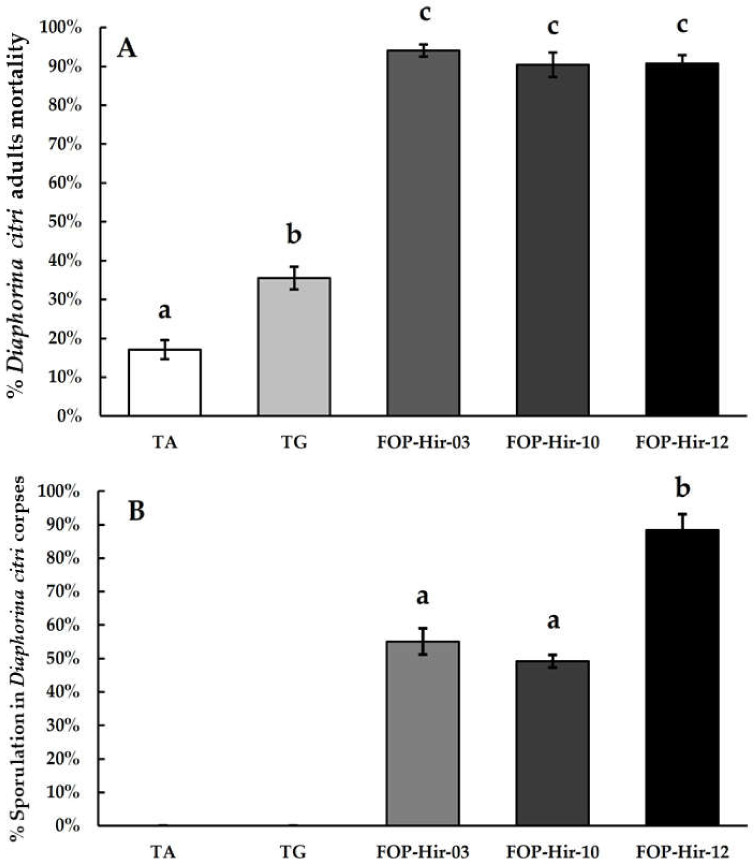
Average percentages of mortality and development of aerial mycelium (sporulation) caused by formulated *Hirsutella citriformis* conidia (5 × 10^6^ conidia/mL) applied via spraying *Diaphorina citri* adults under field conditions. TA = absolute control; TG = control without active agent; FOP-Hir-03 = OP-Hir-3 strain formulation; FOP-Hir-10 = OP-Hir-10 strain formulation; and FOP-Hir-12 = OP-Hir-12 strain formulation. (**A**) Average mortality percentage. Bars indicate the standard error (Tukey α ˂ 0.05), and different lower-case letters indicate a significant statistical difference between treatments. (**B**) Percentage of the average development of aerial mycelium. Different letters indicate a statistically significant (Dunn’s α ˂ 0.05) difference in the percentage of sporulation of the fungus in the infected insects, using the Kruskal–Wallis test.

In a second trial performed in October 2022, following the same treatments and dosages as in the first trial, formulated conidia (5 × 10^6^ conidia/mL) from *H. citriformis* strains caused a mortality of *D. citri* adults ranging from 90.7% to 94.0%, resulting in significantly (F_4,39_ = 85.4; *p* ≤ 0.05) higher mortality compared with that of the control (17.0%), whereas the TG treatment application resulted in 35.4% mortality among *D. citri* adults. In addition, the absolute control showed 1.5% mortality in the March 2022 trial and 17.0% in the October 2022 trial (Figure 4A). *D. citri* cadavers developing aerial mycelia after the application of treatments ranged from 88.4% with the FOP-Hir-12 treatment to 55.0% and 49.1% for FOP-Hir-10 and FOP-Hir-03 treatments, respectively (Figure 4B). 

A significantly (*H* (4) = 37.7; *p* < 0.001) higher proportion of mycosed cadavers were found during the October 2022 bioassay. Among treatments, the formulated OP-Hir-12 strain (FOP-Hir-12) conidia showed the highest percentage of aerial mycelium on *D. citri* adult cadavers, compared to all other treatments.

## 3. Discussion

Epizootics are often caused by pathogenic fungi that naturally control Hemipteran insect populations through horizontal transmission, including *Diaphorina citri*, whose epizootics are mainly due to the fungus *Hirsutella citriformis* Speare [18]. However, few studies, particularly related to this fungus’s distribution, pathogenicity, and biocontrol of *D. citri*, have been reported. This study was undertaken to evaluate the effectiveness of formulated conidia from three selected *H. citriformis* strains and gums at controlling *D. citri* adults. For the pest control of most insects, a growers’ first choice are chemical insecticides. In testing an integrated approach for managing *D. citri*, foliar applications of broad-spectrum insecticides provided short-term (one to two weeks) control of this citrus pest. Unfortunately, it leads to a population suppression of ladybeetles. A long-term suppression (two months) was observed after applying neonicotinoid systemic insecticides by drench. Application of other chemicals did not show an effective control for this insect pest. Previous studies have recommended the application of biological control to enhance the long-term management of this pest [19].

In the present study, we showed that the strains of formulated *H. citriformis* conidia were effective at infecting and killing *D. citri* adults under laboratory, greenhouse, and field conditions. In addition, formulations have shown conditions of maintaining conidia viability for at least 90 d after storage at 25.0 °C and at least 120 d after storage at 4.0 °C [20]. We also demonstrated that *H. citriformis* gum was toxic to *D. citri* adults, as previously reported [17]. Despite the fact that insects were placed in humidity chambers, we did not observe mycelium development in the carcasses of treated insects across the different treatments. 

In this regard, *H. citriformis*-produced gum was toxic to *D. citri* adults (about 49% of the population died after gum exposure) in laboratory, greenhouse, and field experiments. *D. citri* mortality was higher (>50% mortality) under laboratory conditions, after applying the OP-Hir-10 strain (FOP-Hir-10GH) or *Hirsutella* gum (TGH)-formulated conidia, as compared to the absolute control. 

In addition, the LT_50_ was 9 d (±6.2–11.7 d) using the FOP-Hir-10GH treatment and was 14.7 d for the TGH (without conidia) treatment after a 21 d exposure period using 1 × 10^7^ conidia/mL, which agrees with previous studies [11,12,15,21,22]. The insecticidal effect of *Hirsutella* gum may be due to the production of toxins, such as Hirsutellin A (*Ht*A), Hirsutellin B (*Ht*B), phtalic acid, ribotoxins, or some exopolysaccharides that have been reported by several authors as affecting some species of mites and insect larvae [19,23,24,25,26,27].

Results of greenhouse experiments evidenced that FOP-Hir-3, FOP-Hir-10, and FOP-Hir-12 treatments caused 55.7%, 56.1%, and 84.6% *D. citri* mortality, respectively, and the percentage of cadavers showing aerial mycelium was low (18.4% to 38.7%). These study results agreed with those previously reported [1], showing that after applying *Isaria fumosorosea* and *Beauveria bassiana* under greenhouse conditions at the concentration of 1 × 10^8^ conidia/mL, the observed mortality of *D. citri* adults was 72.1% and 61.2%, respectively. Furthermore, the percentage of cadavers with aerial mycelium was 52.6% and 38.4%, respectively, which was probably due to weather conditions (25.7 °C ± 1 °C and 93.0% relative humidity, during the evaluation period) [27]. The optimal development temperature of *H. citriformis* is 25 °C ± 2 °C [19]. Similar results in *D. citri* adults have been observed after applying *B. bassiana* and *M. anisopliae* conidia (1 × 10^7^ conidia/mL), reaching a mortality of 51.0% and 78.9%, respectively [28]. Moreover, *H. citriformis* conidia (5 × 10^6^ conidia/mL) virulence against *D. citri* adults was higher than that of other entomopathogenic fungi, where 70.0% *D. citri* mortality was observed after applying 10^7^ to 10^8^ conidia/mL of *B. bassiana* NCIM 1216, *B. bassiana* 2067, *C. fumosorosea* Wize IF-171201, and *M. anisopliae* 2411 [1,28].

In field experiments, formulations showed a 54.2% to 94.0% mortality of *D. citri* adults, which may be related to a genetic variability of the evaluated strains that is associated with high pathogenicity and virulence, as observed in many pathogens such as *B. bassiana* and *M. anisopliae* [29,30]. We also showed that *Hirsutella* gum killed about 41.2% of adult insects in the field, similar to that observed under laboratory and greenhouse conditions. In the first field experiment, the presence of aerial mycelium on collected cadavers was affected by environmental conditions, mainly by the low temperature of 21.5 °C and relative humidity of 73.5%, since the optimal temperature for *H. citriformis* growth is 25.0 °C [22]. In addition, fungal infections of cadavers by *H. citriformis* have been increasingly reported during the autumn and winter but they are almost absent during spring and summer, probably due to suboptimal relative humidity levels for *H. citriformis* [15]. In the second experiment, the environmental conditions (25.7 °C and 72.5% relative humidity) allowed fungal mycelium to develop in a large percentage of the collected adult cadavers (55.0% to 88.4%). The *D. citri* mortality achieved using formulated *H. citriformis* conidia applied in the field was similar to that previously reported in field trials [19], where *Hirsutella* gum was used in the formulation, causing a mortality of 65.8% to 80.0% in two experiments. 

It was reported that a *H. citriformis* strain conidia, formulated with 1.0% *Acacia* gum and sprayed on *D. citri* adults in a Persian lemon orchard, caused lower mortality from 35.7% to 51.0%, compared to our present results [13], which were also similar to those previously reported [3] in field bioassays, using *Isaria fumosorosea* ESALQ-1296 and *Beauveria bassiana* ESALQ-PL63 at 2.5 and 5 × 10^6^ conidia/mL, respectively, against *D. citri* adults in a commercial citrus orchard. *I. fumosorosea* caused an average mortality rate of 79.5% and 82.5%, respectively, whereas *B. bassiana* caused 76.4% and 82.3% mortality, respectively. However, other authors have reported a mortality rate of *D. citri* adults of 42.0%, 50.0%, and 50.0% after four applications in the field at concentrations of 2 × 10^13^ conidia/hectare of *I. fumosorosea, B. bassiana*, and *M. anisopliae*, respectively [31]. 

The greenhouse and field results from our study showed that the “base formulation” (powdered vegetable oil and gums) is compatible with all tested strains and does not affect their pathogenicity against *D. citri* adults. Furthermore, the absence of adverse effects of *H. citriformis* on predators such as *Chrysoperla rufilabris* Burmeister (Neuroptera:Chrysopidae, and *Hippodamia convergens* Guérin-Méneville (Coleoptera: Coccinellidae), has been reported [32], thus demonstrating that its application in formulations against *D. citri* adults minimizes the impact on beneficial organisms and increases mortality against the pest insect.

## 4. Materials and Methods

### 4.1. Fungal Origin and Preparation 

OP-Hir-3, OP-Hir-10, and OP-Hir-12 *H. citriformis* strains were previously isolated from *Diaphorina citri* adult cadavers, showing fungal aerial mycelia, in Tabasco, Yucatán, and Colima, México, respectively [20]. Strains are available in a public strain collection at the National Center for Biological Control, Mexico (Centro Nacional de Referencia de Control Biológico-CNRCB; accessed by 28 August 2023). Strains were kept in 10.0% glycerol and cultured on potato dextrose agar with 1.0% yeast extract (PDAY; Difco Laboratories, Detroit, MI, USA) at 28.0 °C ± 2.0 °C for six weeks to achieve abundant conidiation. Conidia were produced via solid culturing on oats, and their viability was tested as previously reported [20]. Produced conidia were quantified with a Neubauer chamber (Marienfeld, Germany) and their viability was determined before each experiment. For the bioassays, we only used conidia with at least 90.0% viability.

### 4.2. Production of Hirsutella citriformis Gum

*Hirsutella* gum was produced using the *H. citriformis* OP-Hir-9 strain, which was isolated from *D. citri*-mycosed cadavers collected from Perssé lemon (*Citrus latifolia* L.) in a citrus orchard located in Quintana Roo, México [20]. It was cultured in potato dextrose broth (Difco Laboratories) and PDBY (Difco Laboratories). Media was inoculated with 3.0 cm^2^ agar-grown *H. citriformis* and incubated for 14 d at 25.0 °C ± 2.0 °C and 180 rpm. The resulting produced gum was separated from the fungal culture via centrifugation for 5 min at 10,000 rpm. Gum was collected and mixed with isopropanol and gum (vol:vol) at a ratio of 3 to 1, without shaking for 6 h at 25.0 °C ± 2.0 °C, after which it was dried in an oven (model Heratherm OMH180; Thermo Fisher Scientific, Waltham, MA, USA) at 70.0 °C overnight and crushed in a mortar. *Hirsutella* gum production was processed using six lots from February to June 2023. The resulting gum from all lots was homogenized for conidia formulation or gum-alone treatments.

### 4.3. Hirsutella citriformis Conidia Formulation for Laboratory, Greenhouse, and Field Trials

Formulations were prepared as oil-in-gum emulsions, using conidia from each of the selected strains. To evaluate the compatibility of the components at the laboratory level, formulations were prepared using 0.5% (*w*/*v*) *Acacia* gum and 3.0% (*w*/*v*) powdered vegetable oil, without conidia for the TGA treatment [20]; 0.5% (*w*/*v*) *Acacia* gum, 3.0% (*w*/*v*) powdered vegetable oil, and *H. citriformis* conidia for the FOP-Hir-10GA treatment; and 0.5% (*w*/*v*) *Hirsutella* gum and 3% (*w*/*v*) powdered vegetable oil and *H. citriformis* conidia for the FOP-Hir-10GH treatment. All treatments were sterilized via autoclaving for 15 min at 121 °C, after which they were cooled at room temperature and conidia were added at a final concentration of 1 × 10^7^ conidia/mL. In bioassays, formulations were prepared in the morning and applied during the afternoon to facilitate fungi growth and avoid the loss of viability of conidia.

For greenhouse and field experiments, formulations were prepared by mixing 0.1% *Acacia* gum, 0.4% *Hirsutella* gum, and 3.0% vegetable oil powder in distilled water and vortexing at speed #5 for 20.0 s, which resulted in an oil-in-water emulsion. The emulsion was autoclaved at 121 °C for 15 min and 103 kPa and cooled at room temperature, after which *H. citriformis* conidia from each strain were added at 1 × 10^7^ conidia/mL (final concentration). 

### 4.4. Experimental Insects

For laboratory bioassays, *D. citri* adults were collected from citrus trees in the urban area of Nuevo León, México, in the morning before bioassays. For field bioassays, insects were collected from lemon trees in Tecomán, Colima, México, (*Citrus aurantiifolia*), in the same orchards where bioassays were performed.

### 4.5. Hirsutella citriformis Efficacy against Diaphorina citri Adults under Laboratory Conditions

We evaluated the laboratory effectiveness of one *H. citriformis* conidia formulation (1 × 10^7^ conidia/mL). The additive effect of *Acacia* or *Hirsutella* gums used as adherent to formulate conidia was achieved by adding 3.0% powdered vegetable oil [20]. Bioassays consisted of seven replicates of 15 *D. citri* adults/treatment, including absolute control (TA), *Acacia* gum control (TGA), *Hirsutella* gum control (TGH), conidia formulated with *Acacia* gum and powdered vegetable oil, and conidia formulated with *Hirsutella* gum and powdered vegetable oil [20]. 

Treatments were sprayed directly using one milliliter of each suspension on each group of 15 adult insects in experimental chambers and incubated at 28.0 °C ± 2.0 °C, under a 12 h:12 h photoperiod. Treatments were sprayed in the afternoon before bioassays were performed. The experimental bioassay consisted of 150 mL plastic containers with sand, measuring 6 cm height × 8 cm top × 5 cm bottom, where we placed a two-centimeter-thick sponge layer saturated with distilled water to provide humidity and 15 *D. citri* adults on one leaf [21]. After incubation, insect mortality was recorded every three days for up to 21 d. Bioassays were arranged as randomized experimental designs, testing four replicates per treatment. Data were analyzed to calculate mortality percentage and lethal time to kill 50.0% of the treated population (LT_50_). Percentage mortality was calculated according to the following Abbott’s correction formula [33,34]:

$$\mathrm{Abbott}’s\;\mathrm{correction}=\frac{\left(\%\;\mathrm{treatment}-\%\;\mathrm{absolute}\;\mathrm{control}\right)}{\left(100\%-\%\;\mathrm{absolute}\;\mathrm{control}\right)}\times 100$$

### 4.6. Formulated Hirsutella citriformis Conidia Efficacy against Diaphorina citri Adults under Greenhouse Conditions

Efficacy evaluation of formulated *H. citriformis* conidia was determined inside a greenhouse of the Mexican *Centro Nacional de Referencia de Control Biológico*, Tecomán, Colima (Centro Nacional de Referencia de Control Biológico, Servicio Nacional de Sanidad, Inocuidad y Calidad Agroalimentaria, México; accessed by 28 August 2023), using 12 orange (*C. × sinensis*) plants. This bioassay began on 28 October and ended on 18 November 2022. Five shoots per plant were infested with 10 adults (unsexed, 10 d to 15 d postemergence) per shoot and confined using a mesh bag (5.0 cm height × 28.0 cm width). The infestation of insects on shoots was performed in the morning before bioassays. Treatments included absolute control (TA), *Hirsutella* gum control (TGH), and the formulated *H. citriformis* strains FOP-Hir-3 (Tabasco), FOP-Hir-10 (Yucatan), and FOP-Hir-12 (Colima). Formulated conidia were adjusted to 5 × 10^6^ conidia/mL and 4.0 mL per shoot was applied in the afternoon, using a handheld sprayer. The mortality of *D. citri* adults was evaluated after 21 d by quantifying the number of dead, alive without aerial mycelia, and mycosed adults. Cadaver carcasses were placed in humid chambers (25.0 °C ± 2.0 °C and 90.0% relative humidity) for 10 d to verify aerial mycelia development. Temperature and relative humidity were determined daily by a hygrometer (Elitech RC-4HC datalogger; Elitech Technology Inc., San Jose, CA, USA) placed in the plant canopy. Bioassays consisted of a randomized block experiment with five replicate determinations per treatment.

### 4.7. Efficacy of Formulated Hirsutella citriformis Conidia against Diaphorina citri Adults under Field Conditions

The effectiveness of formulations based on *H. citriformis* against *D. citri* adults was evaluated in two independent field experiments. The first field experiment was developed on a commercial Mexican Persian lemon orchard located in the Tecomán municipality, *Colima* state, Mexico, (18°55′53.2″ N, 103°51′51.6″ O) in 2022 from 23 March to 12 April. The orchard area used for this experiment had a density of 187 three-year-old plants/hectare of *Citrus aurantiifolia* with an average height of two meters. Plants were three meters apart in columns and rows. Conidia from OPG-Hir-3, OPG-11, and OPG-Hir-12 *H. citriformis* strains were produced on oats as a culture substrate and used in the formulation as an active agent [19]. Three formulations were prepared with each of the strains at a concentration of 5 × 10^6^ conidia/mL, in addition to a control without the active agent (TG) and an absolute control (TA). Eight Mexican lemon trees were tested for each treatment, selecting six shoots per tree, where we placed 10 *D. citri* adults, covered in a mesh bag. Each shoot was inoculated via foliar spraying with four milliliters of each treatment solution. After 21 d application, live and dead insects were quantified inside each mesh bag, and cadavers were incubated in a humidity chamber to evaluate the development of aerial mycelia. For each experiment, a hygrometer was placed to record temperature and relative humidity levels every four hours during the experiment period.

The second field bioassay done in 2022 began on 28 October and ended on 18 November. It was also performed in a Mexican lemon (*C. aurantiifolia*) orchard in Tecomán, Colima, México (18°49′07.5″ N 103°48′33.6″ W). The effectiveness of FOP-Hir-3, FOP-Hir-10, and FOP-Hir-12 was evaluated in a similar way as described for the first bioassay, where 10 *D. citri* adults were used on each shoot, six shoots per tree, and eight trees for each treatment. The same three formulations were applied at a concentration of 5 × 10^6^ conidia/mL, including a control without the active agent (TG) and an absolute control (TA). Each shoot was sprayed with four milliliters of formulation. Mortality was determined 21 d post application. Formulation application was performed in the afternoon before bioassays. During the bioassay period, temperature and relative humidity levels were recorded with a hygrometer.

### 4.8. Data Analysis

We determined *D. citri* adults’ mortality after exposure to *H. citriformis* strains. In laboratory bioassays, lethal time data from treatments were calculated using the Kaplan–Meier test. Mortality percentages were transformed with arcsine for normalization. Data were adjusted for control mortality using the Abbott’s correction formula, where the absolute control value was subtracted from all other values [34]. One-way ANOVA was used to analyze the mortality data of bioassays, using the Tukey’s post hoc test at a significance level of *p* ≤ 0.05 (IBM SPSS Statistics Version 21; SPSS, Inc., Chicago, IL, USA). The Kruskal–Wallis test was used to analyze sporulation percentage data from the different bioassays and where analyzed data showed no normality, and was compared using the Dunn’s test, using the Graph Pad Prism 9 software (GraphPad Software Inc., San Diego, CA, USA).

## 5. Conclusions

Our studies demonstrated the potential of *Hirsutella citriformis* strains and gums to control *Diaphorina citri* adults, in addition to demonstrating the bioinsecticide effect of *Hirsutella* gum on these insects in the field. The use of powdered vegetable oil and *Hirsutella* gum are compatible components that do not affect the pathogenicity of *Hirsutella citriformis* conidia.

## Figures and Tables

**Table 1:** *Diaphorina citri* adults’ median lethal time (LT_50_) in days after exposure to formulations under laboratory conditions.

Treatments					
	TA	TGA	TGH	FOP-Hir-10GA	FOP-Hir-10GH
**LT_50_**	19.1 ± 0.6	18.2 ± 0.6	14.7 ± 0.9	18.4 ± 0.7	9.0 ± 1.4
**95% Confidence limit**	(17.9–20.3)	(16.9–19.5)	(12.9–16.5)	(17.0–19.9)	(6.2–11.7)

## Data Availability

Not applicable.
